# Assessment of Three Antimicrobial Residue Concentrations in Broiler Chicken Droppings as a Potential Risk Factor for Public Health and Environment

**DOI:** 10.3390/ijerph16010024

**Published:** 2018-12-21

**Authors:** Karina Yévenes, Ekaterina Pokrant, Fernando Pérez, Ricardo Riquelme, Constanza Avello, Aldo Maddaleno, Betty San Martín, Javiera Cornejo

**Affiliations:** 1 Department of Preventive Medicine, Faculty of Veterinary and Animal Sciences, University of Chile, Av. Santa Rosa, La Pintana, Santiago 11735, Chile; kariyevenescoa@gmail.com (K.Y.); katiavalerievna@ug.uchile.cl (E.P.); fernandocamilo.pm@gmail.com (F.P.); ricardo.riquelme@uchile.cl (R.R.); coniavello@gmail.com (C.A.); 2 Laboratory of Veterinary Pharmacology, Faculty of Veterinary and Animal Sciences, University of Chile, Av. Santa Rosa, La Pintana, Santiago 11735, Chile; amaddaleno@veterinaria.uchile.cl (A.M.); bsmartin@uchile.cl (B.S.M.)

**Keywords:** antimicrobial residues, chlortetracycline, florfenicol, sulfachloropyridazine, chicken droppings, LC-MS/MS

## Abstract

Tetracyclines, sulfonamides and amphenicols are broad spectrum antimicrobial drugs that are widely used in poultry farming. However, a high proportion of these drugs can be excreted at high concentrations in droppings, even after the end of a therapy course. This work intended to assess and compare concentrations of florfenicol (FF), florfenicol amine (FFa), chlortetracycline (CTC), 4-epi-chlortetracycline (4-epi-CTC), and sulfachloropyridazine (SCP) in broiler chicken droppings. To this end, 70 chickens were housed under controlled environmental conditions, and assigned to experimental groups that were treated with therapeutic doses of either 10% FF, 20% CTC, or 10% SCP. Consequently, we implemented and designed an in-house validation for three analytical methodologies, which allowed us to quantify the concentrations of these three antimicrobial drugs using liquid chromatography coupled to mass spectrometry (LC-MS/MS). Our results showed that FF and FFa concentrations were detected in chicken droppings up to day 10 after ceasing treatment, while CTC and 4-epi-CTC were detected up to day 25. As for SCP residues, these were detected up to day 21. Noticeably, CTC showed the longest excretion period, as well as the highest concentrations detected after the end of its administration using therapeutic doses.

## 1. Introduction

Antimicrobials are routinely used to treat clinical livestock diseases, as well as to control common disease events, or even as animal growth promoters [1]. Though the latter use is not allowed in many countries worldwide, in some still continue using them for prophylaxis and as growth promoters’ purposes [2,3]. Tetracyclines and sulfonamides are some of the most frequently used antimicrobial classes in animal production throughout the world [4]. Additionally, drugs from the amphenicol class are used exclusively in the veterinary field, especially florfenicol (FF) [5]. These antimicrobials are extensively used in poultry farming for treating several diseases, such as black head disease, salmonellosis, pullorum disease, coccidiosis, omphalitis, and fowl typhoid disease [6]. According to some reports, it is in poultry farms from developing countries where antimicrobial drugs are being used more heavily [7], with India and Asian countries showing an expected use growth of at least 124% by year 2030 [8].

Some researchers have provided evidence that animals excrete 30 to 90% of antimicrobial drugs after its administration, either as the unmetabolized original drug or as their active metabolites [9,10,11,12]. This evidence agrees with findings from other researchers who reported detecting high concentrations of antimicrobials in faeces from several production animals. In particular, these researchers found significant evidence of tetracyclines, sulfonamides, and amphenicols in droppings from broiler chickens and manure from cattle and swine with drug concentrations that ranged from 1.4 μg to 300 mg of drug per kg, or L of faeces [13,14,15,16,17]. 

As FF and chlortetracycline (CTC) are partially transformed into their main active metabolites—florfenicol amine (FFa) and 4-epi-chlortetracycline (4-epi-CTC), respectively—these are also used as markers for residues of these antimicrobials [18,19]. Contrarily, sulfachloropyridazine (SCP) only undergoes minor metabolisation into N-4-acetylsulfonamide, and this metabolite shows poor antimicrobial activity by itself, hence only the original drug is quantifiable [20].

Broiler litter is comprised of poultry droppings, spilled food, and feathers on top of the actual bedding material, as well as the microbiota that develops within this substrate [21]. Several classes of antimicrobials have been detected in this by-product—especially fluoroquinolones, sulfonamides, and tetracyclines [22]—hence turning litter into an important source of resistant bacteria and resistance genes [23,24,25]. One possible use for these litter is as an ingredient for cattle feeds, which results in passive reintroduction of pathogens and antimicrobial drugs into animals [26,27]. Another widely common use is as organic fertilizers [28,29,30], thus broiler litter has also become one of the main routes for entry of antimicrobials and resistance genes into the environment [31,32,33,34]. These findings are highly significant in light of the estimated 11 and 14 million tonnes of litter reported by big poultry producers, such as Brazil and the United States of America, respectively—usually recycled to use it as organic fertilizers [23,35].

Ongoing release of antimicrobial residues only partially explains their detection in the environment, because it is also a consequence of the prolonged persistence of these drugs in Nature. In particular, tetracyclines are significantly more persistent than other drugs, resulting in higher concentrations in the environment and especially on soils [36,37]. Meanwhile, sulfonamides residues show lower persistence in the environment but some researchers have reported that these migrate down to deeper layers in the soil, reaching aquifers, and some of their metabolites exhibit greater toxicity than the parental drug [36,38]. Similarly, low persistence in the environment has been reported for FF residues, which are degraded by hydrolysis, photolysis, and other mechanisms [39,40]. Such persistence of antimicrobial residues is greatly relevant as some researchers have reported that residues are absorbed and accumulated by crop plant species [41,42].

The presence of these drugs in the environment can result in ecological disturbances and phytotoxicological effects [37,43]. Specifically, some studies have revealed how toxic levels of residues from amphenicols, tetracyclines, and sulfonamides can impact sprouting and growth rates of crop plants [44,45], as well as on growth, reproductive parameters, and lethality of aquatic organisms [46,47,48].

Bearing in mind that antimicrobials are massively used nowadays, as well as its presence in animal faeces, it is clear that this situation becomes a risk factor for both public health and environment—due to the presence of residues in both foods and nature, as well as the possibility of pathogens developing resistance to antimicrobials [12,22]. Consequently, in this work we intended to expand the knowledge regarding the spread of drugs designed for veterinary use. To this end, we administered therapeutic doses of pharmaceutical formulations of FF, CTC, and SCP to groups of broiler chickens, followed by assessing residues concentration for these drugs in their droppings by liquid chromatography coupled to tandem mass spectrometry (HPLC MS/MS). 

## 2. Materials and Methods 

### 2.1. Experimental Animals

Broiler chickens from the Ross^®^ 308 genetic line were used to study each antimicrobial (N = 70 per group) and were housed in battery cages under controlled environment conditions (25 ± 5 °C, 50−60% relative humidity). Birds were provided with *ad libitum* access to water and non-medicated food that was formulated to cover the nutritional requirements recommended for the breed by the genetic company. This food was tested to ensure it was free of antimicrobial residues using an HPLC MS/MS method. 

Two groups of birds were assigned for each antimicrobial drug, one experimental and the other a control group. Experimental groups were treated with commercial formulations and received therapeutic doses of 10% FF (30 mg/kg/day for 5 days), 20% CTC (50 mg/kg/day for 7 days), and 10% SCP (30 mg/kg/day for 5 days). On the other hand, control groups received no antimicrobial drugs, and all of these groups were sampled at seven different times after ceasing treatment with each drug.

The European Medicines Agency recommended some criteria to calculate experimental group sizes, which were deemed appropriate for this work and implemented. These recommendations were detailed in the Note for Guidance EMA/CVMP/SWP/735325/2012 regarding “Approach towards harmonization of withdrawal periods” [49].

Animal housing met all regulations of animal welfare, as approved by the Bioethics Committee from the Faculty of Veterinary and Animal Sciences of the University of Chile, which were outlined in resolution 03-2013 and dated on 11th of November of 2013. These recommendations were based on Directive 2010/63/EU on the protection of animals used for scientific purposes [50].

### 2.2. Collection of Samples

Group samples were collected from dropping boards that were placed below the wire mesh floor of each cage. Different zones of these trays were sampled for each sampling day, and then pooled before storing them at −20 °C within labelled plastic bags while waiting for further processing, analyte extraction and chromatographic analysis.

### 2.3. Quantification 

#### 2.3.1. Reagents

All solvents used in this work were HPLC-grade quality. Standard florfenicol and florfenicol amine solutions of certified purity (99.8%) were used to analyse and quantify those analytes. The Internal Standard (IS) was chloramphenicol-d_5_ (CAF-d_5_) of certified purity (97%). All standards were manufactured by Sigma-Aldrich^®^ (Merck KgaA, Darmstadt, Germany). 

Standard solutions of chlortetracycline-hydrochloride (CTC) and 4-epi-chlortetracycline (4-epi-CTC) of certified purity (95.6%) were used for the analysis and quantification of these analytes. Additionally, isotopic tetracycline-d_6_ (TC-d_6_) of certified purity (>80%) was used for the internal standard. These standards were manufactured by Dr. Ehrenstorfer Gmbh (LGC Standards, Middlesex, UK) and Toronto Research Chemicals (Toronto, ON, Canada), respectively.

A standard solution of SCP of certified purity (99.7%) was used to analyse and quantify residues of this drug, and a standard of sulfamethazine-phenyl-^13^C_6_ hemihydrate (SMZ) of certified purity (99.9%) was used for the internal standard. These standards were manufactured by Sigma-Aldrich^®^ (Merck KgaA, Darmstadt, Germany).

#### 2.3.2. Sample Preparation, Extraction and Clean-Up

**Figure 1:**
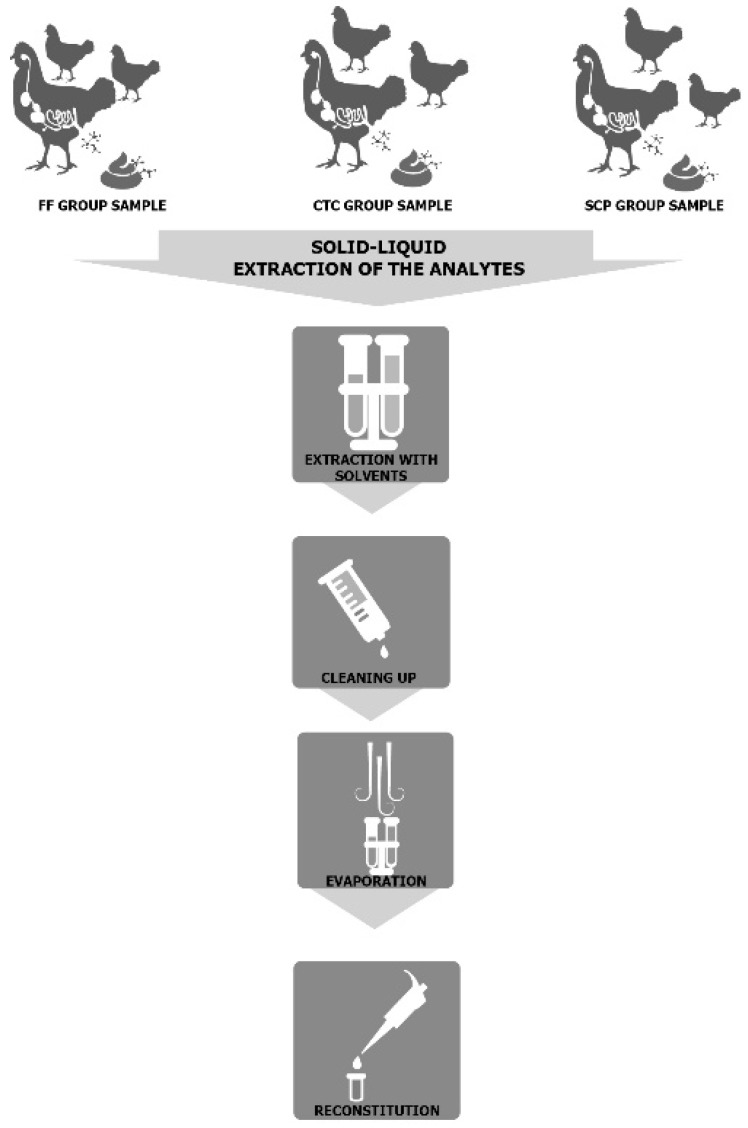
Solid-liquid extraction of FF, CTC, and SCP analytes from group samples of broiler chicken droppings.

All samples were initially homogenized with a spoon before collecting 2 g of droppings in a 50 mL polypropylene tube. Then, every experimental and control sample was spiked with the appropriate internal standard for each antimicrobial drug, before proceeding to extract and clean them (Figure 1).

##### Florfenicol and Florfenicol Amine

In each tube, 10 mL of HPLC water and 10 mL of acetone were added to these samples before they were agitated, sonicated, and centrifuged. The resulting supernatant was sieved through glass wool and 33 mm Millex^®^ filters (Merck & Co., Darmstadt, Germany) with 0.22 µm polyvinylidene fluoride (PVDF) membranes, as it was transferred to another 50 mL polypropylene tube where it was mixed with 7 mL of dichloromethane. Tubes were agitated, sonicated and centrifuged again, and the upper phase of the solution was discarded. Then, OASIS™ HLB^®^ (Waters Corp., Milford, MA, USA) extraction columns were conditioned with 6 mL of an acetone/dichloromethane solution (7:3 ratio) before proceeding to elute the samples with 8 mL of methanol. Afterwards, samples were evaporated under mild nitrogen flow in a water bath (45 ± 5 °C). Finally, samples were reconstituted in 700 μL of a methanol/water solution (7:3 ratio). This methodology was adapted from techniques previously published by other authors [51,52,53].

##### Chlortetracycline and 4-epi-Chlortetracycline

First, 4 mL of EDTA-McIlvaine buffer solution and 1 mL of acetonitrile were added to each sample in a 50 mL polypropylene tube and then agitated, sonicated, and centrifuged. The resulting supernatant was sieved through glass wool while being transferred to another 50 mL polypropylene tube. Then, 13 mL of EDTA-McIlvaine buffer solution were added to this tube, mixed, and passed through OASIS™ HLB^®^ (Waters Corp.) solid phase extraction columns. These columns were previously conditioned with 5 mL of methanol and 5 mL of HPLC-grade water. Afterwards, these columns were eluted using 5 mL of methanol, and the elute was evaporated under mild nitrogen flow in a water bath (45 ± 5 °C). Finally, samples were reconstituted in 200 μL of methanol and 300 μL of HPLC-grade water. This method was adapted from the methodology published by Berendsen et al. [54].

##### Sulfachloropyridazine

First, 10 mL of ethyl acetate and 2 g of sodium sulfate anhydrous were added to each sample in a 50 mL polypropylene tube and then agitated, sonicated, and centrifuged. The resulting supernatant was poured into another 50 mL polypropylene tube and extracted again twice (using 10 mL of ethyl acetate), collecting and pooling all of these supernatants each time. This pool was concentrated down to a volume of 15 mL by evaporating it in a water bath (45 ± 5 °C) under a mild nitrogen flow. Then, the concentrated samples were passed through SCX^®^ (J. T. Baker^®^, Avantor^TM^ Performance Materials, LLC, Center Valley, PA, USA) cationic sulfonic acid solid phase extraction columns that had been previously conditioned with 6 mL of hexane and 6 mL of ethyl acetate. A solution of 10 mL of methanol/ammonia (48.5:1.5 mL ratio) was used for elution. The resulting elute was then evaporated under a mild nitrogen flow in a water bath (45 ± 5 °C) and reconstituted in 500 μL of mobile phase solution (phase A and B in a 15/85 proportion). This methodology was adapted from a method previously published by other authors [55,56,57]. All reconstituted samples, for every antimicrobial drug, were transferred to 1.5 mL Eppendorf tubes, which were centrifuged and sieved through a Millex^®^ filter while being poured into labelled autosampler vials that were read using LC-MS/MS.

#### 2.3.3. Instrumental Analysis

An Agilent^®^ series 1290 liquid chromatography system (Agilent, Santa Clara, CA, USA) coupled to an API 5500 (AB Sciex^®^, Framingham, MA, USA) triple quadrupole mass spectrometer were used to detect and quantify FF and FFa. The analytical column was a Synergi™ 4-μm fusion RP 80 Å, 50 × 2.0 mm (Torrance, CA, USA), and the chromatographic separation used a mobile phase of 0.1% acetic acid in water (phase A) and 0.1% acetic acid in water/methanol (1:9 ratio, phase B). The flow rate was 350 μL min^−1^, the gradient elution was 25% phase solvent A, and 75% phase solvent B, the injection volume was 2 μL, and temperature was set at 37 °C.

Meanwhile, an Agilent^®^ series 3200 liquid chromatograph device coupled to an API 4000 (AB Sciex^®^) triple quadrupole mass spectrometer were used to detect and quantify CTC and 4-epi-CTC. The analytical column was a Sunfire™ C_18_ 3.5 μm, 150 × 2.1 mm (Waters Corp.), and the chromatographic separation used a mobile phase of 0.1% formic acid in water (phase A) and 0.1% formic acid in methanol (phase B). The flow rate was 0.2 μL min^−1^, the gradient elution was 78% phase solvent A, and 22% phase solvent B, the injection volume was 25 μL, and temperature was set at 30 °C.

Lastly, an Agilent^®^ series 1200 liquid chromatograph device coupled to an API 3200 (AB Sciex^®^) triple quadrupole mass spectrometer were used to detect and quantify SCP. The analytical column was a Symmetry™ C_8_ 3.5 μm, 100 × 2.1 mm (Waters Corp.) and the chromatographic separation used a mobile phase of 0.1% formic acid in methanol (phase A) and 0.1% formic acid in water (phase B). The flow rate was 200 μL min^−1^, the gradient elution was 45% phase solvent A, and 55% phase solvent B, the injection volume was 20 μL, and temperature was set at 35 °C.

Finally, the Analyst^®^ version 1.6.2 software package (AB SCIEX) was used for the chromatographic integration of all samples. Table 1 lists the monitored ion masses. 

### 2.4. In-House Validation 

The in-house validation protocol was designed following the recommendations from the European Union Commission Decision 2002/657/EC [58]. To ensure that this methodology was suitable for detecting residues of each antimicrobial drug in chicken droppings, the protocol assessed several performance parameters. In particular, the protocol evaluated specificity, limit of detection (LOD), limit of quantification (LOQ) and curve linearity. For each antimicrobial drug, the limit of detection (LOD) was established based on a signal-to-noise ratio greater than 3:1. (averaged from the results of 20 samples, fortified at the LOD). As for the Limit of Quantification (LOQ), it was determined by calculating the sum of these values with the product of 1.64 times their standard deviation. Lastly, linearity was calculated using blank samples that were fortified using five different and equidistant concentrations.

## 3. Results

### 3.1. In-House Validation of Analytical Methodologies

**Figure 2:**
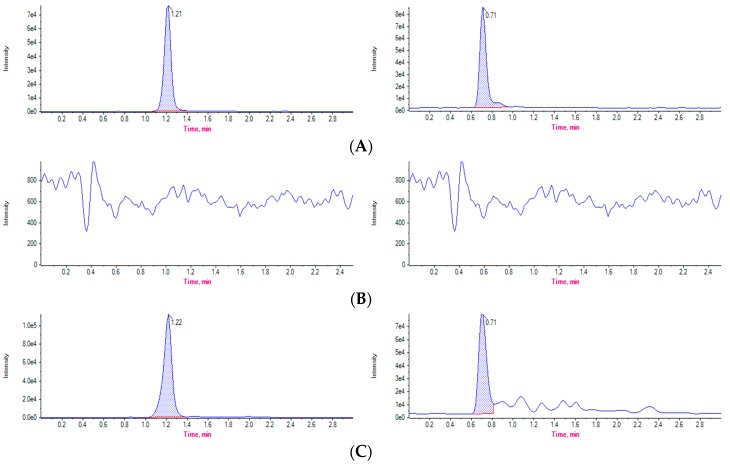
Representative chromatograms from (**A**) Certified standard injection of FF (**left**) and FFa (**right**), (**B**) Blank samples, free of residues of FF (**left**) and FFa (**right**), (**C**) Droppings samples with residues of FF (**left**) and FFa (**right**), from experimental animals at day 10 after ceasing treatment (sampling point 2).

In regard to specificity, we observed no interference from blank samples within the interest region where elution of the analyte is expected (Figure 2). Specifically, the LOD for FF, FFa, CTC, 4-epi-CTC, and SCP were of 50, 50, 20, 20, and 10 μg/kg, respectively. Meanwhile, the LOQ was set at 52.2, 60.5, 22.5, 22.9, and 12.6 μg/kg for FF, FFa, CTC, 4-epi-CTC, and SCP, respectively. As for the calibration curves, these were fortified at concentrations of 50, 100, 200, 300 and 500 μg/kg for FF and FFa; 20, 40, 60, 80 and 100 μg/kg for CTC and 4-Epi-CTC; and 10, 20, 40, 80 and 100 μg/kg for SCP. All of these curves showed a linear response and their R² was greater than 0.99.

### 3.2. Assessment of Antimicrobial Concentrations in Broiler Chicken Droppings

#### 3.2.1. Detection and Quantification of FF and FFa in Broiler Chicken Droppings

Quantification results showed that FF and FFa concentrations declined by 76% in droppings between days 5 and 10 after ceasing treatment, down to a final concentration of 136.23 μg/kg on the second sampling point (see Figure 2). After day 10, no residues were detected. Table 2 details concentrations of FF and FFa detected for each sampling point after ceasing antimicrobial treatment.

#### 3.2.2. Detection and Quantification of CTC and 4-epi-CTC in Broiler Chicken Droppings

CTC and 4-epi-CTC residues were detected at every sampling point. These concentrations declined steadily over time, up to day 18 after ceasing treatment, which was the inflexion point when residue concentrations began rising up again and reaching a value of 179.45 μg/kg at the seventh sampling point. Table 3 lists these concentrations as detected for each sampling point. 

#### 3.2.3. Detection and Quantification of SCP in Broiler Chicken Droppings

SCP residues in droppings declined steadily at each sampling point and were detected only up to day 21 after ceasing treatment. In particular, concentrations decreased by 94% between days 2 and 21, reaching a final value of 24.261 μg/kg on the fourth sampling point. Table 4 details concentrations of SCP detected for each sampling point after ceasing antimicrobial treatment.

## 4. Discussion

Lately, faeces of production animals have become an important analytical matrix, mostly due to the fact that high concentrations of the antimicrobials commonly administered can be excreted by this route [12]. Therefore, analyzing these drugs in faeces might become an important tool to collect more information in regard to the development of antimicrobial resistance within the intestine of animals and about the dissemination of antimicrobials in the environment, as well as also helping on the effort to monitor the usage trends for antimicrobial drugs, whether it is legally, illegally, or off-label [59].

The finding of FF residues up to day 10 after ceasing treatment might be a consequence of its pharmacokinetic properties, such as its fast and efficient absorption by the gastrointestinal system, its large volume of distribution, and its good tissue penetration [60,61]. Not long ago, [62] identified FF and FFa residues in broiler chicken feathers up to day 40 after ceasing treatment, detecting concentrations that exceeded 100 μg/kg. Similarly, [63] detected them in chicken claws up to day 25 after ceasing treatment. Thus, the fact that these residues could be identified in our work up to day 10 after ceasing treatment might be a consequence of such prolonged bioaccumulation within feathers and claws, as well as the rapid excretion of these metabolites [64].

As for SCP, residue concentrations in droppings decreased steadily up to day 21 after ceasing treatment, reaching values of 24.261 μg/kg of droppings. Meanwhile, a study found that SCP residues could be detected in feathers up to day 36 after ceasing treatment [65], stating that this drug can bioaccumulate for extended periods in feathers of broiler chickens—a phenomenon that correlates with its wide distribution over peripheral tissues [66]—and that could explain why these residues could only be detected up to day 21 after ceasing treatment. Additionally, pharmacokinetic properties could explain why the excretion period is so narrow for SCP: they are mainly eliminated by the renal route, metabolites are excreted faster than the unmetabolized drug, and the low urinary pH favours their reabsorption, hence prolonging their excretion half-lives [6,67].

Faecal excretion of CTC might be attributed to it undergoing an entero-hepatic cycle, where the drug is reabsorbed from the intestine after its excretion in the bile. Several important changes can be noticed when a significant fraction of the drug enters this kind of recirculation mechanism, such as an extension of its half-life and the elimination period via the renal route. Moreover, CTC is excreted mainly by kidneys and follows a slow process [6,68]. Likewise, some studies propose that CTC is eliminated more slowly in broiler chickens than other analogous tetracyclines [18].

We are currently not aware of any study reporting on CTC and 4-epi-CTC depletion in feathers, claws or edible tissues in broiler chickens who received therapeutic doses of this antimicrobial. However, one study detected OTC and 4-epi-OTC concentrations in claws from broiler chickens up to day 19 after ceasing treatment [69]. Similarly, the same research group found residue concentrations of these analytes in feathers up to day 46 after ceasing treatment [70]. Consequently, systemic recirculation of CTC residues from claws and feather might explain the increase observed on CTC and 4-epi-CTC concentrations on days 21 and 25 after ceasing treatment. Nonetheless, in spite of the similarity between the chemical structures of OTC and CTC, some studies in humans and animals suggest that CTC is excreted differently, hence assuming that absorption, distribution, and excretion are similar between different animal species might be an oversimplification [18]. Therefore, to clarify whether the increase in CTC and 4-epi-CTC over the last days was a consequence from systemic recirculation, we should study CTC depletion in several organs of chickens, as well as measuring plasma concentrations of its metabolites. These results were also reported before by our research group in Cornejo et al. [71].

All group samples were thoroughly homogenized to ensure they were appropriately representative of the experimental birds. However, despite it must be consider that this represent a limitation for the study as it does not allow the calculation of a group mean and standard deviation over time, pooling samples offered a global perspective about residue concentrations when birds receive therapeutic doses of antimicrobial drugs.

As the main component of broiler litter is chicken droppings, this by-product presents a potential danger as it might reintroduce antimicrobial residues in the food chain and the environment—especially when used as an organic fertilizer. Furthermore, faeces are stored at room temperature before using them as organic fertilizers, and this impacts differently the persistence of antimicrobial drugs depending on drug class and kind of faeces [59]. These authors also state that in the case of broiler droppings, SCP is slightly persistent, unlike CTC, which persists for much longer periods showing a DT_50_ and a DT_90_ of 18 and 61 days, respectively.

Based on the results observed in this work, CTC could impact public health and the environment more severely than the other antimicrobials we studied, as it is excreted for a longer period and at higher concentrations following a therapeutic dose. Also, CTC is widely used both in veterinary and human medicine, it shows a moderate persistence within organic fertilizers during storage, as well as greater stability, persistence, and toxicity in the environment than other drugs [1,37,43,59]. Evidently then, the potential consequences of its use in animal farming could be alarming.

## 5. Conclusions

The LC-MS/MS methods we implemented and in-house validated for this work allowed us, in a reliable and specific manner, to detect and quantify FF, FFa, CTC, 4-epi-CTC, and SCP analytes in droppings from broiler chickens. Our data showed that these birds excreted these drugs up to days 10 for FF and FFa, day 21 for SCP, and day 25 for CTC and 4-epi-CTC. Overall, CTC would be the antimicrobial drug that poses a greater risk to public health and the environment.

This study is a pioneering work in regard to determining antimicrobial residues in faeces from animals that have been treated therapeutically, thus it contributes significantly to an informed decision-making process that may lead to regulations intended for managing these drugs rationally within the context of animal farming, as well as the practice of using animal faeces as a source of organic fertilizers for crops of human interest.

## Figures and Tables

**Table 1:** Monitored ion masses.

Analyte	Precursor Ion (Da)	Fragment Ion (Da)
FF	356.0	336.0
FFa	248.0	230.0
CAF-d_5_	326.0	157.0
CTC	479.0	444.0
4 epi-CTC	479.0	154.0
TC-d_6_	451.0	416.0
SCP	284.9	155.9
SMZ	285.1	124.1

**Table 2:** FF and FFa residue concentrations in chicken broiler droppings after treatment with a commercial formulation, by sampling point.

Sampling Point	Days after Ceasing Treatment	Age (Days)	Final Concentration of FF + FFa (μg/Kg)
1	5	15	568.35
2	10	20	136.23
3	15	25	<LOD
4	20	30	<LOD
5	25	35	<LOD
6	30	40	<LOD
7	35	45	<LOD

**Table 3:** CTC and 4-epi-CTC in droppings from broiler chickens treated with a commercial formulation, by sampling point.

Sampling Point	Days after Ceasing Treatment	Age (Days)	Final Concentration of CTC + 4-epi-CTC (μg/Kg)
1	5	25	665.82
2	8	28	368.17
3	11	31	258.4
4	15	35	136.88
5	18	38	106.47
6	21	41	112.01
7	25	45	179.45

**Table 4:** SCP concentration in droppings from broiler chickens who were treated with a commercial formulation, by sampling point.

Sampling Point	Days after Ceasing Treatment	Age (Days)	SCP Concentration (μg/Kg)
1	2	11	382.242
2	5	14	124.748
3	10	19	38.186
4	21	30	24.261
5	32	41	<LOD
6	34	43	<LOD
7	36	45	<LOD
